# The organizational social context of mental health services and clinician attitudes toward evidence-based practice: a United States national study

**DOI:** 10.1186/1748-5908-7-56

**Published:** 2012-06-22

**Authors:** Gregory A Aarons, Charles Glisson, Phillip D Green, Kimberly Hoagwood, Kelly J Kelleher, John A Landsverk

**Affiliations:** 1 University of California, San Diego, Department of Psychiatry, 9500 Gilman Drive (0812), La Jolla, CA, 92093-0812, USA; 2 Child and Adolescent Services Research Center at Rady Children’s Hospital San Diego, 3020 Children’s Way MC-5033, San Diego, CA, 92132, USA; 3 Children's Mental Health Services Research Center, Henson Hall, University of Tennessee, Knoxville, TN, 37996, USA; 4 New York University School of Medicine, One Park Avenue at East 33rd, 8th Floor, New York, NY, 10016, USA; 5 New York State Office of Mental Health, 44 Holland Avenue, Albany, NY, 12229, USA; 6 The Ohio State University, Pediatrics and Public Health, 700 Children’s Drive, Columbus, OH, 43205, USA

**Keywords:** EBPAS, OSC, Evidence-based practice, Evidence-based practice attitude scale, Mental health services, Provider attitudes, Organizational social context, Culture, Climate

## Abstract

**Background:**

Evidence-based practices have not been routinely adopted in community mental health organizations despite the support of scientific evidence and in some cases even legislative or regulatory action. We examined the association of clinician attitudes toward evidence-based practice with organizational culture, climate, and other characteristics in a nationally representative sample of mental health organizations in the United States.

**Methods:**

In-person, group-administered surveys were conducted with a sample of 1,112 mental health service providers in a nationwide sample of 100 mental health service institutions in 26 states in the United States. The study examines these associations with a two-level Hierarchical Linear Modeling (HLM) analysis of responses to the Evidence-Based Practice Attitude Scale (EBPAS) at the individual clinician level as a function of the Organizational Social Context (OSC) measure at the organizational level, controlling for other organization and clinician characteristics.

**Results:**

We found that more proficient organizational cultures and more engaged and less stressful organizational climates were associated with positive clinician attitudes toward adopting evidence-based practice.

**Conclusions:**

The findings suggest that organizational intervention strategies for improving the organizational social context of mental health services may contribute to the success of evidence-based practice dissemination and implementation efforts by influencing clinician attitudes.

## Background

Multiple factors at system, organizational, and individual levels influence the implementation of evidence-based practices (EBPs) and other innovations in public sector social service and mental healthcare settings [1-4]. These factors include policy, social and economic characteristics, characteristics of the innovation itself, characteristics of the organization attempting to implement the innovation, and characteristics of service providers and clients [1,3-8]. One of the most proximal influences on service providers’ attitudes and behaviors is the social context of the organizations in which they work [9]. This paper examines the association of organizational social context, other organizational factors, and clinician demographics with clinician attitudes toward adopting EBPs in a United States national sample of mental health clinicians.

The role of organizational social context in the development of individual-level work attitudes and behavior has been the focus of organizational research for several decades. This long line of research has established that work-related attitudes of individuals in a variety of different types of work environments are influenced by the organizational social contexts in which they work [5,10-12]. Organizational social context includes the norms and expectations (i.e., culture) of the organization for its members as well as the psychological impact of the work environment on the individual workers (i.e., climate). Culture and climate are related, complex, multidimensional constructs, but there is evidence that they are distinct and that each affects work attitudes in unique ways [13]. Culture is expected to influence the work attitudes of individual workers through their accommodation to the expectations (e.g., an emphasis on proficiency) that govern their day-to-day work behavior. Climate is expected to influence work attitudes through the psychological impact of the work environment (e.g., the level of stress) on the workers.

While the vast majority of research on culture and climate has been conducted in business and industrial organizations, there is a growing interest in the cultures and climates of healthcare, mental health, social service, and other human service organizations. While few controlled studies have examined actual organization context change efforts [14], research provides evidence that culture, climate, and work attitudes compose an organizational social context that is associated with worker turnover, new program sustainability, service quality, and service outcomes [9,13,15-20]. Here, we examine variance in mental health clinicians’ attitudes towards EBPs explained by specific dimensions of organizational culture and climate in order to better understand the role that organizational social context might play in complementing or inhibiting efforts to disseminate and implement EBPs.

Mental health clinicians have varying and complex attitudes toward EBPs [21,22]. While an overall positive or negative view of EBPs may be held by clinicians, clinicians can also simultaneously hold somewhat contradictory attitudes. That is, clinicians can be positively predisposed to EBPs on one dimension and negatively predisposed on another. For example, the idea that the practices clinicians use in providing mental healthcare should be informed by empirical evidence might be appealing to some clinicians who might also be unwilling to implement specific EBPs that are required by their employer or by a state mental health agency [23]. Or, clinicians may be open to learning more about EBPs, but at the same time believe that what they have learned in the world of practice experience is much more salient to selecting appropriate psychotherapeutic approaches than evidence provided by published research [23].

Aarons et al. [21,23,24] captured this complexity in the Evidence-Based Practice Attitude Scale (EBPAS) and documented four distinct dimensions in clinicians’ attitudes toward EBPs. The four dimensions include the intuitive appeal of EBPs, the likelihood of adopting EBPs as a result of institutional requirements, the perceived divergence between research-based practices and current practice, and general openness to learning new practices. Moreover, they found that certain clinician characteristics at the individual level (e.g., years of experience, education level) influence each of these various dimensions differently. For example, clinicians with more advanced degrees described EBPs as more appealing but at the same time were less willing to implement EBPs simply because they were required.

EBPAS scores have been associated with provider characteristics related to implementing new service technologies (See Aarons et al., 2012 for a review [25]). For example, female service providers have higher scores on the Appeal [26,27] and Requirements subscales [26] as well as higher total EBPAS scores [26]. Higher educational attainment has been associated with higher scores on the Appeal subscale [23,26-28] and lower scores on the Requirements subscale [26]. Aarons [23] found no difference in EBPAS scores by discipline (e.g., social work, psychology), but a later study found that providers with their highest degree in social work had higher Openness subscale scores and higher Total EBPAS scores than those with their highest degree in psychology. Clinicians with a degree in psychology, however, endorsed lower perceived Divergence between EBP and usual practice compared to clinicians with a degree in social work [26].

EBPAS scores have also been associated with knowledge and use of EBPs in other healthcare disciplines and settings. In a study of physicians, more positive EBPAS scores were associated with higher levels of physician knowledge of technological innovations supporting evidence-based clinical practice [29]. In a study of implementation of Parent–child Interaction Therapy (PCIT), more positive scores on Appeal were related to increased clinician attendance in PCIT consultation, while lower perceived Divergence between EBP and usual care was associated with increased phone coaching [30]. In substance abuse treatment settings, more positive attitudes toward EBP have been demonstrated to predict self-reported use of evidence-based cognitive/motivational treatment approaches [31] and adoption of contingency management, an evidence-based substance abuse treatment approach [32].

Studies examining transfer of learning in varied settings and types of health practice have also found relationships between attitudes and EBP. One study found that occupational therapy students in Ireland endorsed willingness to practice EBP [33]. Another study found that while medical residents in Japan endorsed generally positive attitudes toward EBP, few could articulate the construct or were familiar with the use of standard research sources in medical decision making [34]. However, a survey of physicians in the United Kingdom found a greater prevalence of positive attitudes toward EBP and greater ability to articulate elements of evidence-based medicine [35]. Thus, while studies across countries and healthcare practice settings have indicated variability in attitudes and knowledge related to EBP, none have examined the link between organizational context and attitudes in nationally representative samples.

Studies have indicated that change in organizational culture and climate in healthcare settings is a complex proposition that must take into account the impacts of autonomy and influence that occur in complex healthcare organizations [36,37]. In addition, individually held attitudes constitute but one factor in a complex context that may influence adoption and use of EBP [6]. Still, providing conceptual linkages between system and organizational context, provider attitudes and characteristics, and adoption and use of EBP is needed to help understand and develop strategies to improve EBP implementation and sustainment.

The organizational social contexts of providers’ work environments are also related to providers’ attitudes toward EBP. Positive climate measured by the Organizational Readiness for Change scale was negatively related to Divergence scores on the EBPAS [38], and demoralizing or negative organizational climate has been found to be positively related to Divergence scores [15]. These results indicate that providers in organizations with positive climates perceive less Divergence between EBP and their usual practice, while those in organizations with poor climates perceive more Divergence. An organizational culture that promotes achievement and mutual encouragement has been positively associated with higher scores on Appeal and Openness subscales, as well as higher total EBPAS scores, while an organizational culture emphasizing rules and orders (i.e., ‘command and control’) over personal beliefs and ideas, has been associated with higher Divergence scores [15]. Finally, mental health providers working for private non-profit organizations endorsed more positive attitudes toward EBP relative to public sector mental health departments [39].

Previous studies linking organizational social context and attitudes toward EBPs were conducted with limited samples and fewer organizational context dimensions, so further validation with more representative samples using multidimensional measures of both organizational culture and climate is needed. The present study builds on previous work by examining the association of attitudes toward EBP as measured by the EBPAS [23,26], with multiple dimensions of organizational social context as measured by the Organizational Social Context (OSC) scales [16] in a nationally representative sample of mental health agencies from all regions of the United States.

We developed several hypotheses based on the extant literature. By including predictors at both organizational and individual levels in a multi-level analysis, it is possible to partition the variance in attitudes explained by factors at each level. This is important because we know that culture and climate characteristics at the organizational level are not completely independent from individual clinician level characteristics. For example, organizational policies and leader behaviors can signal to providers the important cultural values of the organization [40]. Therefore, analyses at a single level—whether the organizational level or individual level—could confound the association of attitudes toward EBPs with variables at the other level. In addition, it is important to consider the independent and combined roles played by multiple dimensions of culture and climate simultaneously in explaining different dimensions of clinicians’ (often conflicted) attitudes toward EBPs. Thus, we hypothesized that variance in attitudes toward EBPs would be explained by variables at both the organizational and individual levels.

Organizational culture and climate are each expected to influence attitudes about EBPs in different ways. Organizational culture captures the expectations and values about what is important in a specific organization. These expectations and values, either implicitly or explicitly expressed in the behavior of fellow workers, have the capacity to socialize members of the organizations who may seek to behave in ways that meet the expectations of their workplace [9]. As a result, culture should influence attitudes about EBPs in ways that comply with organizational expectations. For example, clinicians who work in cultures that expect organizational members to be proficient in their work (i.e., have up-to-date knowledge, be effective in their work) should have more positive views of EBPs. Thus, we hypothesized that providers working in organizations with proficient cultures would have more positive overall attitudes toward EBPs as evidenced by higher scores on the EBPAS total scale score. In addition, we hypothesized that more proficient cultures would be associated with higher scores on the openness, appeal, and requirements scales, and lower scores on the divergence scale. We hypothesized that clinicians working in organizations with rigid or resistant cultures would be more likely to have lower EBPAS total, Appeal, Openness and Requirements scores, and higher Divergence Scores.

Organizational climate is expected to influence attitudes about EBPs somewhat differently. Climate describes the psychological impact of the work environment on clinicians (e.g., stress) [9]. Therefore, although an organization might expect its members to be proficient, clinicians in high-stress work environments might feel that requirements imposed on them by implementing new EBPs are overwhelming. Thus, we hypothesized that providers working in organizations with more stressful climates would endorse more negative attitudes toward EBPs as evidenced by lower scores on the Appeal, Openness, and Requirements scales, and higher scores on the Divergence scale. We also hypothesized that clinicians working in organizations in more functional climates would report more positive attitudes toward EBP. Finally, we hypothesized that clinicians working in organizations with engaged climates would endorse higher scores on the Appeal scale because this scale emphasizes the importance of colleague perceptions and practicality of EBP.

## Methods

The national onsite, in-person survey of mental health clinicians who provided mental health services in the sampled clinics is one of two surveys composing the Clinical Systems Project (CSP) of the Research Network on Children’s Mental Health funded by the John D. and Catherine T. MacArthur Foundation [16]. The other survey was a telephone survey of mental health clinic directors [41]. The sampling frame for these surveys began with the counties selected for the National Survey of Child and Adolescent Well-being (described in [42]). The sample of 100 clinics participating in the clinician survey represents clinics from the CSP that met minimum size criteria (five or more clinicians) and whose director agreed to an onsite survey of clinicians in a scheduled staff meeting. Comparisons of the characteristics of clinics represented in the clinic directors survey that did and did not meet these criteria show similar characteristics in the number of clinicians employed in the clinic, clinician turnover rates, and the proportions of clinicians who are psychiatrists, Ph.D. psychologists, and MSW social workers. However, a slightly higher proportion of therapists in the clinics that participated in the clinician survey were BSW social workers (13% versus 8%).

### Participants

Trained external research assistants who had no formal working relationships with the clinics conducted the onsite surveys in person with clinicians who treated either children or both children and adults in each of the 100 participating mental health clinics. Respondents in each clinic completed the surveys simultaneously during a scheduled staff meeting with no upper managers present, after receiving assurances of confidentiality from the external research assistant. The respondents returned the surveys at the end of the meeting directly to the research assistant using sealed envelopes. In some clinics, more than one meeting was necessary to obtain data from all clinicians. The response rate for the clinicians per site ranged from 30% to 100% with an overall average response rate of 76%.

### Clinician demographics

Demographics were assessed through clinician self-report of age, gender, race/ethnicity, educational level, major of highest degree, years of experience, and job tenure. As shown in Table 1, 1,112 clinicians from 100 clinics participated in 75 cities in 26 states from all parts of the United States. Among the respondents, 24% worked for public mental health agencies, 2% for private for-profit agencies, and 74% for private not-for-profit agencies. As shown in Table 2, the age of the participating clinicians by clinic ranged from 21 to 74 years, and clinicians had up to 50 years of experience with a mean of 11 years of experience. Most of the clinicians were female (76%) and Caucasian (71%), and had masters degrees (67%), with majors in either social work (41%) or psychology (32%). In addition, smaller proportions held doctorates (7%) and were either African American (15%) or Hispanic (7%).

**Table 1:** Number and location of clinics

	**Clinics**	**Clinicians**
Participating Clinicians		1,112
Clinics	100	
Midwest	31	
South	28	
Northeast	22	
West	19	
Cities	75	
Counties	61	
States	26	

**Table 2:** Characteristics of clinicians

	**Percent**	**Min.**	**Max.**	**Mean**	**SD**
Number of clinicians per clinic		3	52	11.54	8.32
Age of participants		21	74	38.26	11.48
Years of experience		0	50.0	10.76	8.55
Years in agency		0	35.0	4.36	4.96
Gender					
Male	23.6				
Female	76.4				
Ethnicity					
African American	15.1				
Asian American	2.0				
Caucasian	70.7				
Hispanic	7.3				
Native American	0.3				
Other	4.7				
Educational level					
High School	0.5				
Some college	2.2				
Bachelors degree	15.9				
Some graduate work	7.0				
Masters degree	67.4				
Doctoral degree	7.1				
Major of highest degree					
Education	4.8				
Medicine	1.1				
Nursing	0.7				
Psychology	31.8				
Social Work	40.9				
Other	20.7				

Note: N = 1,112; Min. = Minimum; Max. = Maximum; SD = Standard Deviation.

### Measures

#### Evidence-Based Practice Attitudes Scale (EBPAS)

The EBPAS consists of 15 items measured on a 5-point Likert scale ranging from 0 (not at all) to 4 (to a very great extent) [23,24,26]. The EBPAS is conceptualized as consisting of four lower-order factors/subscales and a higher-order factor or total score that represents the respondent’s global attitude toward the use of EBPs [26]. Previous studies suggest moderate to good internal consistency reliability in two samples (Cronbach’s alpha total scale ranging from 0.77 to 0.79, subscales ranging from 0.59 to 0.93) [23,24]. Construct validity is supported by two previous scale development studies [23,24] and by studies of associations between EBPAS and mental health clinic structure and policies [23], culture and climate [15] and leadership [27].

Scores for each subscale were calculated for the present analyses by summing the items composing each subscale. A Total Scale Score was calculated by summing all 15 items after reverse coding the items composing the Divergence Scale. The alpha reliability for measuring the Total Score was 0.76 in this sample.

Among the four lower-order factors that compose the Total Score, the Appeal Score assesses the extent to which the provider would adopt an EBP if it were intuitively appealing, could be used correctly, or was being used by colleagues who were happy with it. The alpha reliability for measuring Appeal was 0.80 in this sample. The Requirements Score indicates the extent to which the provider would adopt an EBP if it were required by an agency, supervisor, or state. The alpha reliability for measuring Requirements was 0.91 in this sample. The Openness Score indicates the extent to which the provider is generally open to trying new interventions and would be willing to try or use more structured or manualized interventions. The alpha for measuring Openness was 0.84 in this sample. The Divergence Score indicates the extent to which the provider perceives EBPs as not useful and less important than clinical experience. The alpha reliability for measuring Divergence was 0.66 in this sample.

#### Organizational Social Context (OSC)

The OSC includes 105 items that measure seven primary scales that were developed in multiple validity and reliability studies over a period of three decades to assess three domains of the social context of mental health and social service organizations: Organizational Culture, Organizational Climate, and Work Attitudes [16]. The OSC was completed by individual clinicians in staff meetings as described above and the scale scores were aggregated by clinic site after confirming agreement among clinicians within each clinic [16]. The OSC measures of culture and climate are included in the present analyses and described below.

Organizational Culture is defined as the expectations that govern how work is done in an organization and the OSC assesses organizational culture on three second-order dimensions: rigidity, proficiency, and resistance. Previous studies have provided evidence of the validity and reliability of these scales and associated organizational culture with individualized care, service quality, staff turnover, staff attitudes toward their work, productivity, efficiency, and the sustainability of new treatment programs in a variety of mental health and social service organizations [5,12,19,43-46].

A Rigid Organizational Culture is characterized by expectations that clinicians will have little discretion or flexibility in carrying out their jobs, provide limited input into key management decisions, and carefully follow a host of bureaucratic rules and regulations. This dimension is assessed with items measuring centralization (e.g., ‘I have to ask a supervisor or coordinator before I do almost anything’) and formalization (e.g., ‘the same steps must be followed in processing every piece of work’). The alpha reliability for measuring Rigidity in this sample is 0.81.

A Proficient Organizational Culture is characterized by expectations that clinicians will place the well-being of each client first and that clinicians will be competent and have up-to-date knowledge. Proficient cultures expect clinicians to be both skilled and attentive to the needs of individual clients. Proficiency is assessed with items measuring responsiveness (e.g., ‘members of my organizational unit are expected to be responsive to the needs of each client’) and competence (e.g., ‘members of my organizational unit are expected to have up-to-date knowledge’). The alpha reliability for measuring Proficiency in this sample is 0.94.

A Resistant Organizational Culture is characterized by expectations that clinicians will show little interest in change or in new ways of providing service, and that clinicians will suppress any interest in change with criticism and apathy. Resistance is assessed with items measuring apathy (e.g., ‘members of my organizational unit are expected to not make waves’) and suppression (e.g., ‘members of my organizational unit are expected to be critical’). The alpha reliability for measuring Resistance in this sample is 0.81.

Organizational Climate is defined as the employees’ perceptions of the psychological impact of the work environment on their own well-being and functioning in the organization [47]. An organizational climate is formed when employees in the same organizational unit share similar perceptions about the psychological impact of their work environment. The OSC measures climate on three second-order factors: engagement, functionality, and stress. Previous studies have provided evidence of the validity and reliability of these scales and associated organizational climate with service quality, staff turnover, job satisfaction and commitment, and service outcomes in a variety of mental health and social service organizations [5,12,19,20,45,48].

An Engaged Climate is characterized by employee perceptions that they are able to personally accomplish many worthwhile things, remain personally involved in their work and sustain concern about their clients. Engagement is assessed with items measuring personalization (e.g., ‘I feel I treat some of the clients I serve as impersonal objects’ – reverse coded) and personal accomplishment (e.g., ‘I have accomplished many worthwhile things in this job’). The alpha reliability for measuring Engagement in this sample is 0.78.

A Functional Climate is characterized by employee perceptions that they receive the cooperation and help they need from coworkers and administrators to do a good job, have opportunities for personal advancement and growth, and have a clear understanding of how they fit in, and can work successfully within the organization. Functionality is assessed with items measuring growth and advancement (e.g., ‘this agency provides numerous opportunities to advance if you work for it’), role clarity (e.g., ‘my job responsibilities are clearly defined’), and cooperation (e.g., ‘there is a feeling of cooperation among my coworkers’). The alpha reliability for measuring Functionality in this sample is 0.90.

A Stressful Climate is characterized by employee perceptions that they are emotionally exhausted from their work, overloaded in their work, and unable to get the necessary things done. Stress is assessed with items measuring emotional exhaustion (e.g., ‘I feel like I am at the end of my rope’), role conflict (e.g., ‘interests of the clients are often replaced by bureaucratic concerns, e.g., paperwork’), and role overload (e.g., ‘the amount of work I have to do keeps me from doing a good job’). The alpha reliability for measuring Stressful Climate in this sample is 0.94.

#### Analyses

Pearson product–moment correlations were computed at the individual level for responses to the OSC scales assessing culture and climate and the EBPAS scales assessing attitudes toward EBPs. The clinic response rate was also included in the correlation matrix to assess whether the response rate by clinic was correlated with clinician scale responses. In addition, the level of agreement of clinician responses to each culture and climate scale were assessed for each clinic using the r_wg_ coefficient [45].

Each of the EBPAS scales (the total score and the four subscales) were regressed on predictors in two-level random regression models (individuals nested within organizations) using HLM 6 statistical modeling software [49]. For each analysis, an unconditional model was estimated first including only the intercept to compute the intraclass correlation (ICC) and organizational variance for each EBPAS scale. In the second analysis, individual-level, clinician characteristics and group centered responses to the culture and climate measures were added in the first level. Organization-level characteristics, including measures of organizational culture and climate assessed as the mean responses of clinicians in each clinic, were added in the second level. This strategy allowed the between-organizational differences in the EBPAS scales to be distinguished from the within-organizational level differences in EBPAS scales for each unit difference in the OSC scales at the organization and individual levels, respectively.

In addition to estimating regression coefficients for each predictor, the effects of categorical variables at the clinic level (e.g., type of agency) and clinician level (e.g., race/ethnicity) were assessed using a model comparison approach, based on likelihood ratio tests of nested models. This was calculated as the difference in deviance statistics (−2 log likelihood) between the full model and the model without the contrasts (parameters) that compose the particular categorical variable of interest [49]. The significance of the difference in deviance scores is tested as a chi-square value with the degrees of freedom equal to the number of contrasts composing the variable.

## Results

The correlation matrix in Table 3 shows that the clinic response rate (i.e., the proportion of clinicians completing scales within each clinic) is unrelated to the OSC or EBPAS scale values, as indicated by correlations that range from −0.05 to 0.03 (all non-significant). The pattern of correlations within the matrix also suggests that the responses to the OSC and EBPAS are not explained by common method error variance. The size of the correlations between scales from the two instruments (OSC and EBPAS) range from a low of 0.00 (p < 0.990) for rigid culture and EBPAS total to a high of 0.24 (p < 0.000) for proficient culture and EBPAS total. Moreover, despite the statistical power of the large sample (n = 1,112), only half of the 35 correlations between the two sets of scales are statistically significant. Among the dimensions of culture and climate, proficient culture is the most highly correlated with attitudes toward EBPs. Significant correlations include the relationships between proficient culture and the EBPAS Total (0.24, p < 0.001), Requirements (0.19, p < 0.001), Appeal (0.20, p < 0.001), and Openness (0.20, p < 0.001).

**Table 3:** Correlation matrix for Clinic Response Rate, EBPAS, and OSC Scales (n = 1,112)

**Variable**	**1**	**2**	**3**	**4**	**5**	**6**	**7**	**8**	**9**	**10**	**11**	**12**
1. Clinic Response Rate	---											
2. EBPAS Total	−0.03	---										
3. EBPAS Requirements	−0.02	0.65^***^	---									
4. EBPAS Appeal	0.01	0.73^***^	0.34	---								
5. EBPAS Openness	−0.05	0.72^***^	0.25	0.48	---							
6. EBPAS Divergence	0.02	−0.47^***^	−0.06	−0.08^*^	−0.11^***^	---						
7. Proficiency	−0.03	0.24^***^	0.19^***^	0.20^***^	0.20^***^	−0.05	---					
8. Rigidity	0.03	0.00	0.05	−0.02	0.05	0.11^***^	−0.12^***^	---				
9. Resistance	0.03	−0.04	0.02	−0.03	0.07^*^	0.19^***^	−0.31^***^	0.44^***^	---			
10. Engagement	−0.05	0.16^***^	0.06^*^	0.18^***^	0.15^***^	−0.02	0.32^***^	−0.16^***^	−0.24^***^	---		
11. Functionality	−0.01	0.14^***^	0.14^***^	0.10^***^	0.08^**^	−0.04	0.51^***^	−0.27^***^	−0.40^***^	0.35^***^	---	
12. Stress	0.03	−0.02	0.07^*^	0.06	0.09^**^	0.14^***^	−0.27^***^	0.38^***^	0.49^***^	−0.34^***^	−0.54^***^	---

^*^ p < 0.05, ^**^ p < 0.01, ^***^ p < 0.001.

The within clinic agreement in clinician descriptions of their clinic’s culture and climate was assessed with r_wg_[45]. The average r_wg_ for each scale ranged between 0.91 and 0.96 for each clinic. The lowest r_wg_ values for each scale across all clinics ranged between 0.58 and 0.87, and the highest values for each scale ranged between 0.98 and 0.99. These statistics represent strong within clinic agreement among clinicians completing the OSC and support the aggregation of those responses for clinic level measures of organizational culture and climate.

Intraclass correlation coefficients (ICCs) in Tables 4 and 5 provide an estimate of the degree of clustering of responses to the EBPAS scales within groups (represented here by mental health clinics). Although ICCs are typically small in relation to estimates of the proportion of between group to total variation provided by an ANOVA-based eta-squared coefficient, the effect of clustering with ICCs as low as 0.01 to 0.05 can be dramatic on estimates using ordinary least squares regression that assumes ICC = 0.00 [50]. The ICCs reported in Tables 4 and 5 for the five EBPAS scales range between 0.02 to 0.06, and four of the five are statistically significant as reported in Table 5, indicating that attitudes about EBPs are clustered by clinic and thus need random regression models that account for the clustering. The ICCs and eta-squared coefficients (which range from 0.11 to 0.15) reported in Tables 4 and 5 confirm that a significant proportion of the variance in clinicians’ attitudes toward the use of EBPs is a function of the clinic in which the clinician works. Tables 4 and 5 also show the proportions of the organizational-level or clinic variance in attitudes toward EBPs that is explained by organizational social context and by all organizational and individual predictors combined.

**Table 4:** Hierarchical linear model analysis of total EBPAS total score

**Predictor**	** *χ* **^**2**^ (** *df* **)^**a**^	**b**	** *SE* **	**95% CI**	** *T* **	** *df* **	** *p* **
Intercept		36.45 ^***^	6.19	[48.75, 24.15]	5.89	88	0.000
Organizational Level							
Rigid Culture		0.01	0.11	[0.23, -0.21]	0.09	88	0.933
Proficient Culture		0.23 ^*^	0.11	[0.45, 0.01]	2.09	88	0.039
Resistant Culture		0.12	0.12	[0.36, -0.12]	1.00	88	0.320
Stressful Climate		−0.03	0.04	[0.05, -0.11]	−0.77	88	0.442
Engaged Climate		0.21	0.14	[0.49, -0.07]	1.49	88	0.140
Functional Climate		−0.14	0.09	[0.04, -0.32]	−1.47	88	0.146
Type of agency (reference group = public)	13.62^**^ (2)						
For Profit		−4.79	2.49	[0.16, -9.74]	−1.93	88	0.057
Non Profit		1.04	0.78	[2.59, -0.51]	1.32	88	0.190
Service Structure (reference group = separate child division)	1.78 (2)						
Child and Adult		0.19	0.82	[1.82, -1.44]	0.23	88	0.822
Child Only		−0.23	0.35	[0.47, -0.93]	−0.67	88	0.506
Individual Level							
Female		0.81	0.58	[1.95, -0.33]	1.39	923	0.162
Education Level		0.10	0.31	[0.71, -0.51]	0.33	923	0.739
Years Experience		−0.18 ^***^	0.03	[−0.12, -0.24]	−7.39	923	0.000
Rigid Culture	0.02	0.04	[0.10, -0.06]	0.38	923	0.701	
Proficient Culture	0.13 ^***^	0.04	[0.21, 0.05]	3.85	923	0.000	
Resistant Culture	0.05	0.04	[0.13, -0.03]	1.41	923	0.158	
Stressful Climate	0.05 ^*^	0.02	[0.09, 0.01]	2.30	923	0.022	
Engaged Climate	0.19 ^***^	0.05	[0.29, 0.09]	3.81	923	0.000	
Functional Climate	0.10 ^***^	0.03	[0.16, 0.04]	3.31	923	0.001	
Race/Ethnicity (reference group = Caucasian)	177.94^***^ (5)						
African American		−2.94 ^***^	0.78	[−1.41, -4.47]	−3.78	923	0.000
Asian		1.68	1.83	[5.27, -1.91]	0.91	923	0.361
Hispanic		−0.97	0.98	[0.95, -2.89]	−0.98	923	0.326
Native American		4.22	6.56	[17.09, -8.65]	0.64	923	0.520
Other Race		−2.72 ^**^	1.03	[−0.70, -4.74]	−2.63	923	0.009
Major (reference group = psychology)	40.63^***^ (5)						
Education		−0.01	0.92	[1.80, -1.82]	−0.01	923	0.990
Medical		−0.41	1.64	[2.81, -3.63]	−0.25	923	0.804
Nursing		−0.89	2.55	[4.11, -5.89]	−0.35	923	0.726
Social Work		1.77 ^**^	0.58	[2.91, 0.63]	3.08	923	0.003
Other		−0.59	0.60	[0.59, -1.77]	−0.98	923	0.329
ICC (unconditional) = 0.06							
Eta squared = 0.15^***^							
Organizational Variance Explained by Climate and Culture = 8.2%							
Organizational Variance Explained by Full Model = 28.4%							

Note: N = 1154; EBPAS = Evidence-Based Practice Attitude Scale; b = regression coefficient; *SE* = standard error; *df* = degrees of freedom; *p* = two-tailed significance level; OSC = Organizational Social Context; ICC = Intraclass correlation; ^*^ p < 0.05, ^**^ p < 0.01, ^***^ p < 0.001.

Note: ^a^ Chi Square for each categorical variable calculated as difference in deviance statistics between models with and without parameters representing contrasts between categories that define the variable.

**Table 5:** Hierarchical linear model analyses of EBPAS total scale and subscales

**Predictor**	**Regression Weights and (Chi Square Values **^**a**^)				
	Total	Appeal	Requirements	Openness	Divergence
Intercept	36.45^***^	11.17 ^***^	10.77 ^**^	8.19 ^**^	18.16 ^***^
Organizational Level					
Rigid Culture	0.01	−0.05	0.03	−0.01	−0.04
Proficient Culture	0.23 ^*^	0.04	0.07	0.07	−0.07 ^*^
Resistant Culture	0.12	0.04	0.05	0.03	−0.05
Stressful Climate	−0.03	0.02	−0.04 ^*^	0.02	0.02
Engaged Climate	0.21	0.15 ^**^	−0.07	0.05	−0.08
Functional Climate	−0.14	−0.06	−0.04	−0.02	0.02
Type of agency (reference group = public)	(13.62^***^)	(10.75^**^)	(5.49)	(13.34^**^)	(4.44)
For Profit	−4.79	−2.30 ^*^	−1.47 ^***^	−2.35	−1.19 ^***^
Non Profit	1.04	0.06	0.24	0.43	−0.34 ^*^
Service Structure (reference group = separate child division)	(1.78)	(<0.00)	(<0.00)	(<0.00)	(<0.00)
Child and Adult	0.19	0.17	0.16	−0.24	−0.04
Child Only	−0.23	−0.01	0.02	−0.20	0.05
Individual Level					
Female	0.81	0.09	0.59 ^**^	−0.22	−0.30
Education Level	0.10	0.38 ^**^	−0.22	0.09	0.13
Years Experience	−0.18 ^***^	−0.05 ^***^	−0.04 ^**^	−0.06 ^***^	0.05 ^***^
Rigid Culture	0.00	0.00	0.03	0.00	0.02
Proficient Culture	0.13 ^***^	0.04 ^**^	0.04 ^***^	0.06 ^***^	−0.00
Resistant Culture	0.05	0.03	0.05 ^**^	0.04 ^***^	0.07 ^***^
Stressful Climate	0.05 ^*^	0.03 ^***^	−0.00	0.04 ^***^	0.02 ^*^
Engaged Climate	0.19 ^***^	0.09 ^***^	0.02	0.10 ^***^	0.01
Functional Climate	0.10 ^***^	0.05 ^***^	0.05 ^***^	0.04 ^**^	0.04 ^**^
Race/Ethnicity (reference group - Caucasian)	(177.94^***^)	(148.17^***^)	(118.66^***^)	(107.83^***^)	(117.24^***^)
African American	−2.94 ^***^	−1.50 ^***^	−0.66 ^*^	0.07	0.86 ^**^
Asian	1.68	0.48	0.50	0.59	−0.04
Hispanic	−0.97	−0.91 ^**^	−0.55	0.27	−0.40
Native American	4.22	0.72	0.76	2.63	−0.04
Other Race	−2.72 ^**^	−0.97 ^*^	−0.88	0.023	0.68
Major (reference group = psychology)	(40.63 ^***^)	(23.78^***^)	(16.82^**^)	(30.73^***^)	(13.83^*^)
Education	−0.01	0.14	−0.06	0.17	0.20
Medical	−0.41	−1.38 ^*^	0.45	−0.13	−0.62
Nursing	−0.89	−1.14	−1.00	−0.68	−2.09 ^***^
Social Work	1.77 ^**^	0.47 ^*^	0.36	0.79 ^***^	−0.18
Other	−0.59	−0.05	−0.09	−0.027	0.17
ICC (unconditional)	0.06^***^	0.05^***^	0.04^***^	0.05^**^	0.02
Eta squared	0.15^***^	0.13^**^	0.13^***^	0.13^**^	0.11
Organizational Variance Explained by Climate and Culture	8.2%	35.9%	6.0%	2.8%	85.8%
Organizational Variance Explained by Full Model	28.4%	51.8%	8.7%	42.0%	88.4%

^*^ p < 0.05, ^**^ p < 0.01, ^***^ p < 0.001.

Note: ^a^ Chi Square for each categorical variable calculated as difference in deviance statistics between models with and without parameters representing contrasts between categories that define the variable.

The correlations in Table 3 provided evidence that the relationship between clinician-level responses to the scales measuring proficient culture and the total EBPAS was stronger than the relationship between any other pair of OSC and EBPAS dimensions. The higher order analysis in Table 4 provides further support for our hypothesis that clinicians who work in organizations with proficient cultures have more positive attitudes toward the use of EBPs. The results shown in Table 4 describe a significant unique positive effect of proficient culture on attitudes toward EBPs after controlling for other dimensions of organizational culture, dimensions of organizational climate, type of agency, service structure, clinician level perceptions of culture and climate, and other clinician characteristics (e.g., age, experience, education, et al.). Clinicians who work in clinics that expect them to place the well-being of each client first, to be competent and to have up-to-date knowledge are more likely to have more favorable views toward using EBPs than clinicians who work in clinics that do not have those expectations, regardless of their own demographic characteristics, experience, or training.

It’s important to note that this result was obtained after controlling for type of agency (for profit, non-profit, or public), clinic service structure (child and adult, child only, or separate child division), and clinician gender, education level, years of experience, race, profession (education, medicine, nursing, social work, other or psychology) and individual level responses to the culture and climate scales. Although not shown here, the same association between the clinicians’ attitudes toward EBP and their organizational culture was confirmed without the additional covariates in the analysis.

The HLM model shown above was repeated with each of the EBPAS first order scales (Appeal, Requirements, Openness, Divergence) and is shown in Table 5. Clinicians in clinics with proficient cultures report higher overall EBPAS Total and lower Divergence scores. In contrast, Organizational Climate has a stronger association with the EBPAS Appeal scale. In more engaged climates (where clinicians share the perception that they are able to personally accomplish many worthwhile things, remain personally involved in their work, and sustain concern about their clients), clinicians indicate they would be more likely to adopt an EBP if it were intuitively appealing, could be used correctly, or was being used by colleagues who were pleased with it. Clinicians in more stressful climates (those work environments where clinicians shared the perception that they are emotionally exhausted from their work, overloaded in their work, and unable to get the necessary things done at work) report that they would not be as likely to use an EBP if it were required by their agency, supervisor, or state. This suggests that formal requirements for using EBPs would not be as successful in organizational climates characterized by high levels of stress and that other, or additional implementation strategies should be considered.

The analyses also show that the service structure of the clinic was not associated with the clinicians’ attitudes toward EBPs but the type of agency did play a role in three of the five EBPAS scales (Total, Appeal and Openness). Clinicians in private for profit clinics generally had more negative attitudes toward the use of EBPs.

As shown in Tables 4 and 5, the dimensions of organizational culture and climate together explained the highest proportion of organizational variance in the EBPAS dimensions of Appeal and Openness. In addition, culture and climate together also accounted for the majority of the organizational variance that was explained by the full model in these two criteria and in the EBPAS Requirements scale.

At the individual level, several clinician characteristics were associated with attitudes toward the use of EBPs with years of experience, race/ethnicity and academic major having consistent associations across all five EBPAS scales. Clinicians with more years of experience and African American clinicians reported more negative attitudes toward EBPs after controlling of all other characteristics at the clinician and clinic level. Although the effects of academic major were not entirely consistent across all dimensions, social workers generally held the most positive views of EBPs as indicated by three of the five EBPAS scales.

## Discussion

The important finding in this study is that mental health clinic organizational culture and climate are associated with clinician’s attitudes toward adopting EBPs, even when controlling for the effects of a variety of organizational-level and individual-level characteristics. Clinicians working in organizations characterized by proficient cultures, engaged climates, and less stressful climates have more positive attitudes toward the use of EBPs, but each dimension of culture and climate is uniquely associated with a different dimension of the clinicians’ attitudes. The best unique predictor among the culture and climate dimensions of clinicians having positive overall attitudes toward EBPs is working in a proficient organizational culture. Clinicians in proficient cultures (i.e., the organization expects them to place the well-being of the clients first, to be competent, and have up-to-date knowledge) endorsed more positive overall attitudes toward adopting EBPs and lower perceived divergence between ‘in the trenches’ clinical work and the use of EBPs.

Two dimensions of organizational climate were associated with clinician attitudes toward EBPs, but each was associated with a different attitudinal dimension. Clinicians working in clinics with more stressful climates were less likely to adopt an EBP if it was required by a supervisor, agency, or state. This means clinicians working in settings with high levels of emotional exhaustion and role overload are less likely to respond favorably to policy or regulatory mandates requiring the use of EBPs.

Clinicians working in engaged organizational climates where there is a sense of personal accomplishment, personal involvement in and concern for clients reported a greater likelihood of adopting an EBP if it fit their views of clinical practice and their ability to effectively learn and use (i.e., to have high self-efficacy) a given EBP.

Given our conservative approach of testing hypothesized relationships after controlling for the effects of all other dimensions and predictors at the organizational and individual levels, a number of our hypotheses were not confirmed. The HLM analyses provided tests of the unique effects of each dimension of culture and climate at the organizational level after controlling for the effects of all other culture and climate dimensions and all other predictors at both the organizational and individual levels. This is important because all dimensions of culture and climate were related to at least one dimension of the EBPAS in the zero-order correlations shown in Table 3. Moreover, all dimensions of culture and climate at either the organizational or individual level were related to one or more dimensions of the EBPAS in the HLM analysis. However, our hypotheses and findings focus on the independent association of each of the organizational level dimensions of culture and climate with individual level attitudes toward EBP.

In regard to organizational culture, there were no significant organizational level findings related to rigid or resistant cultures (when controlling for the expectations associated with proficient cultures, the three climate dimensions, and all individual-level predictors). Rigid and resistant cultures were associated with attitudes toward EBP at the zero order level, but these relationships were attenuated when the other dimensions of culture and climate were included in the analyses.

Some of our climate related hypotheses were also not supported. While stressful and engaged climates were associated with poorer attitudes toward adopting EBP, our findings illustrated that only two EBPAS subscales were related to climate. Moreover, although functional climates were associated with EBPAS total, Requirements, Appeal, and Openness scales at the zero-order level, all of these relationships were not statistically significant when other dimensions of culture and climate were included at the organizational and individual levels.

It is possible that additional effects could be found if we utilized profile analysis to characterize clinics by culture/climate profiles (e.g., depicting the levels of culture and climate dimensions in relation to each other) rather than assessing the unique contributions of individual culture and climate dimensions after controlling for the others. That is, there may be profiles of positive and negative culture paired with positive or negative climate that provide a more complex characterization of organizations and are associated with EBP attitudes. Thus, future studies should examine profiles of organizational context that may be associated with attitudes and adoption and use of EBPs.

While organizational culture and climate and attitudes toward EBP have been explored in a previous study [15], the present study is the first to focus on these aspects of the complex relationship between multiple dimensions of social context and attitudes. However, it should be noted that the previous study utilized an earlier measure of organizational context with some overlap with the present measure of organizational social context. The present study advances this work by utilizing a more refined measure of organizational social context validated in a United States nationally representative sample of mental healthcare providers. In particular, the conceptualization of the culture and climate dimensions have been honed to be most relevant to health and allied health service settings and predictive of clinical outcomes and EBP implementation [50].

These findings suggest that the successful dissemination and implementation of EBPs among mental health service providers can be facilitated by the support of strategies for developing positive organizational cultures and climates that might otherwise present barriers to adoption and implementation [50]. In addition to implementation of clinical interventions, broad changes are now occurring in behavioral health settings with significant incentives to deploy accountability systems tied to monitoring and reimbursement. Such initiatives may impact organizational context or conversely be affected by organizational context. Changing organizational culture and climate in mental health and social service systems to prepare for implementation requires strategic, well-planned, and evidence-based strategies specifically designed for those systems [1,40,51]. Some promising developments in this regard are being tested in such organizations using randomized controlled trials, showing that organizational interventions can create improvements not only in organizational social context, but also in the implementation of EBPs and client outcomes [50,52]. In related work, effective leadership and coordinated organizational strategies to develop a positive work environment have been suggested to promote strategic cultures and climates that support the implementation and use of EBPs [40,53,54].

Beyond organizational culture and climate, we identified other factors associated with attitudes toward EBPs that are important to understanding barriers to dissemination and implementation. The service structure of the clinic was not associated with clinician attitudes toward EBPs but the type of agency did play a role. Clinicians working in private for-profit clinics reported more negative attitudes toward the use of EBPs. The finding that clinicians working in private for-profit clinics reported more negative attitudes toward EBPs highlights the complexity of supporting EBP implementation and use. private for-profit clinics generally operate under different financing and reimbursement structures and processes such as fee-for-service. This is in contrast to private non-profits that may rely more heavily on contracts and grants to provide funding for services. The links between organization type and attitudes toward EBPs was explored in another study that found that clinicians working inprivate non-profit clinics, compared to public sector clinics, had more positive attitudes toward EBPs [39]. The authors attributed the finding to the more bureaucratic, hierarchical, and less flexible nature of programs that are part of large public sector mental health authorities.

At the individual level and consistent with previous studies, we found that female clinicians and social workers were more positive in their assessments of the use of EBPs while clinicians with more years of experience were more negative in their assessments. Further research is needed to determine why these characteristics impact attitudes and how to take advantage of what is being done in psychology, social work, and other academic and applied mental health, substance abuse, and social service training programs to promote the adoption and use of EPBs.

The implementation of EBPs is a particularly critical change effort that can improve the quality and outcomes of mental health services. In light of recent healthcare policy change under the Patient Protection and Affordable Care Act it is likely that the landscape of health services in the United States will also change. For example, mental health and physical health services may be more integrated in the same service settings, and federally qualified health centers (FQHCs) are likely to be implementing EBPs at an accelerated pace. Understanding the impact of organizational social context on staff readiness and willingness to implement EBPs in light of these changes can suggest ways to improve implementation. Finally, changes in healthcare policy and the financing of behavioral health, and the reorganization of the systems delivering these services (i.e., primary care and specialty mental health payment and provider networks) are likely to affect both attitudes towards EBPs (may reinforce positive attitudes or not) and organizational processes (may produce different values, may increase stress and burnout, may privilege proficient organizations) that are central to successful implementation.

A number of innovations being adopted by mental health authorities expand the definition of EBP beyond efficacious treatment models. Some examples include continuous data monitoring systems, alerts to target prescribing practices, business engineering to target high volume service users, and client outcomes monitoring and feedback to clinicians. Some of these may be considered to be evidence-based if they are proven to improve outcomes. The EBP referent for the EBPAS is more traditional psychological or psychosocial treatment that has research support and may be manualized and structured. Thus, attitudes towards therapies, clinical interventions, and treatments may be a special case of EBPs tapped by the EBPAS. Further research is needed to better understand the role of social context in attitudes toward a variety of other technical innovations as described above. Attitudes toward clinical practices and treatments may not reflect attitudes toward such innovations. The first author is currently piloting an adapted version of the EBPAS to be used to assess attitudes toward adopting specific innovations or practices.

### Limitations

Some limitations of the present work should be noted. First, we assessed four established domains of attitudes toward EBP. Recent work that occurred after data collection for this study identified an additional eight domains of attitudes for EBP including: EBP limitations, fit with clinical practice, impact of fidelity monitoring, balance of art and science in psychotherapy, workplace burden, impact of job security, perceived organizational support, and the impact of getting feedback on job and clinical performance [21]. Thus, further work is needed to establish the associations of these additional scales with individual differences and organizational social context. Second, while our sample is representative of clinicians working in community-based mental health clinics, our findings may not generalize to clinicians in individual practice or to sectors other than mental health (e.g., alcohol/drug, social services). However, there are a number of similarities across public sectors that would support the impact of common organizational processes for implementation [1].

We recognize that determinants of clinician behavior are multifaceted. In the present study, we examined attitudes toward EBP and attitudes are but one among a group of determinants affecting whether someone will or will not engage in a behavior. Other determinants include social norms (some of which are captured by the OSC) and networks [55,56], self-efficacy [57], locus of control and behavioral intentions [58], expectancies, habits, and environmental constraints. Further, changing behavior might also be facilitated by maximizing the fit of an innovation or practice with the needs of the clinic, and needs of clients [59]. While attitudes are associated with organizational social context dimensions, changing clinician behavior will require strategic attention to potential mediators and moderators of the link between attitudes and behavior change. Conversely, it may be the case that engaging in a behavior such as continued use of an EBP until familiarity is developed, using a data monitoring system to track specific indicators of change, or attending learning collaborative meetings with one’s peers, may change attitudes and beliefs about EBPs. That is, behavioral activation itself may influence attitudes [60].

Although a number of covariates were included in the model to control for confounding effects at both the organizational and individual levels, the observed association between organizational social context and individual attitudes toward EBPs provides no evidence of a causal relationship. To better understand the causal links between context and attitudes, we are currently conducting randomized controlled studies with organizational interventions that focus on improving both social context and the use of EBPs as a way of linking changes in social context at the organizational level with the adoption and implementation of EBPs by clinicians.

Finally, it is important to note that utilizing quantitative measures alone presents challenges in adequately measuring and describing a construct as complex as social context. The need for mixed-methods in understanding the impacts of EBP implementation is emerging as important and critical for a more comprehensive and balanced understanding of implementation process and outcomes [61,62]. In addition, the need for multilevel conceptual approaches and analysis is critical to understanding the complexity of outer and inner contexts of services in healthcare and allied healthcare settings [1,63,64].

## Conclusions

The present study adds to the research base demonstrating that organizational social context is associated with clinician attitudes toward the adoption and use of EBPs [50,65]. While focusing on the organization is only one aspect of the landscape of EBP implementation [1], it is an important one for a number of reasons. First, most EBPs are implemented within organizations and organizational characteristics such as social context predict provider attitudes toward adopting EBP, even when accounting for individual-level covariates. Second, organizational social context is malleable. That is, randomized controlled studies have documented that change in organizational culture and climate, as well as associated improvements in EBP implementation, can be accomplished with targeted improvement strategies [40,50-52,66]. Third, we know that the availability of clinical training and funding may be a necessary but not sufficient condition for successful EBP implementation and that successful implementation strategies must address the context in which EBPs are offered [1,4]. In conclusion, this paper adds to the growing evidence that organizational social context must be addressed in efforts to provide effective mental health treatment services through implementation of the most effective treatments. By improving the organizational social context for EBP we not only improve the work environments for clinicians, but also support the implementation of effective clinical services that decrease symptoms and improve functioning and quality of life of those who are served.

## Competing interests

Gregory A. Aarons is an Associate Editor of *Implementation Science*. The authors declare that they have no other competing interests.

## Authors’ contributions

All authors were involved in the design of the research study. GAA and CG conceptualized the specific research questions and analytic approach for this manuscript. Analyses were conducted by CG and PG. All authors wrote, edited, and revised portions of the manuscript. The MacArthur Research Network on Youth Mental participated in the conceptualization and research design of the study. All authors read and approved the final manuscript.
